# Model-Data Hybrid-Driven Wideband Channel Estimation for Beamspace Massive MIMO Systems

**DOI:** 10.3390/e28020154

**Published:** 2026-01-30

**Authors:** Yang Nie, Zhenghuan Ma, Lili Jing

**Affiliations:** 1 School of Physics and Electronic Information Engineering, Jining Normal University, Ulanqab 012000, China; 2 Institute of Intelligent Signal Processing, Jining Normal University, Ulanqab 012000, China

**Keywords:** model-data, beamspace channel estimation, millimeter-wave, massive MIMO

## Abstract

Accurate channel estimation is critical for enabling effective directional beamforming and spectrally efficient transmission in beamspace massive multiple-input multiple-output (MIMO) systems. However, conventional model-driven algorithms are derived from idealized mathematical models and typically suffer severe performance degradation under model mismatches caused by complex and nonideal propagation environments. Although data-driven deep learning (DL) approaches can learn channel characteristics from data, they typically require large-scale training datasets and demonstrate limited generalization capability. To overcome these limitations, we propose a model-data hybrid-driven network (MD-HDN) scheme to address the wideband beamspace channel estimation problem. In the MD-HDN scheme, we unfold the vector approximate message passing (VAMP) algorithm into a trainable network, where a novel shrinkage function is introduced to enhance the estimation accuracy. Extensive numerical results confirm that the proposed MD-HDN scheme can significantly outperform existing schemes under various signal-to-noise ratio (SNR), and achieve substantial improvements in both estimation accuracy and robustness.

## 1. Introduction

### 1.1. Background

With the large-scale commercialization of fifth-generation (5G) mobile communications, global industries and governments are increasingly shifting their attention to sixth-generation (6G) mobile communications to support a variety of emerging concepts and innovative applications [1]. Millimeter-wave (mmWave) communications are widely regarded as a potential technology in future 6G cellular networks, as they can provide gigabit-per-second (Gbps) data rates and gigahertz (GHz) bandwidths by leveraging abundant spectrum resources [2,3]. However, mmWave signals experience severe free-space path loss that increases with carrier frequency [4]. To mitigate severe path loss, massive multiple-input multiple-output (Massive MIMO) was introduced to provide sufficient array gain [5]. Nevertheless, the conventional Massive MIMO systems allocate a dedicated radio frequency (RF) chain for each antenna, which results in high hardware overhead and power consumption [6].

To improve energy efficiency, mmWave Massive MIMO based on discrete lens antenna arrays (LAAs), namely, beamspace Massive MIMO [7], has garnered considerable interest from industry and academia. By selecting only a small number of power-focused beams, beamspace Massive MIMO considerably reduces the number of RF chains [8]. Nevertheless, since the RF chains are considerably fewer than the physical antennas, acquiring accurate channel state information (CSI) is extremely challenging for beamspace Massive MIMO systems. In addition, considering the beam-squint phenomenon of wideband Massive MIMO systems [9], beamspace channel estimation becomes more complex and difficult to address directly.

### 1.2. Related Works

There now exist several schemes that focus on the beamspace channel estimation [10,11,12,13,14,15]. In [10], a low-complexity beam selection-based channel estimation scheme was introduced, where all beams were first scanned and only a few high-power beams were retained using the power-based classifier. The classical least squares (LS) method was then adopted to perform channel estimation. Building upon this work [10], an efficient two-way training-based method was presented to reduce beam training overhead in [11], thereby significantly decreasing the number of required RF chains. However, the pilot overhead in [10,11] is increased proportionally to the number of antennas, which results in limited resources for data transmission. In order to reduce the pilot overhead, several support detection (SD)-based schemes have been proposed [12,13,14]. In [12], the SD-based scheme was designed to effectively reduce pilot overhead while maintaining satisfactory estimation accuracy. For three-dimensional (3D) beamspace scenarios, an adaptive SD-based scheme was proposed in [13], where the 3D beamspace channel was decomposed into a series of components detected according to their power levels. In [14], by leveraging quasi-orthogonal pilots, a novel SD-based scheme was employed to further lower the required number of RF chains. In addition, a cosparse image reconstruction algorithm at a low signal-to-noise ratio (SNR) was proposed to enhance the performance [15].

However, the aforementioned schemes [10,11,12,13,14,15] are designed for narrowband systems, whereas practical mmWave MIMO systems typically operate over wideband frequency bands to satisfy high-throughput transmission. To this end, several efficient methods have been developed for the wideband beamspace channel [16,17,18]. Specifically, ref. [16] employed the simultaneous orthogonal matching pursuit (SOMP) algorithm to address the channel estimation task by assuming common support in the multiple-measurement vectors (MMV) framework. However, the common-support assumption was limited in the wideband scenarios due to the beam-squint phenomenon [9]. To address this limitation, the authors in [17] employed the successive support detection (SSD) algorithm without assuming common support. In this scheme, the channel was decomposed into individual path components and each component was then estimated separately by exploiting its unique frequency-dependent sparse structure. In addition, the authors in [18] developed a two-stage wideband channel estimation method. In this approach, the angles-of-arrival (AoAs) were initially extracted, and the path gains were then optimized to facilitate the reconstruction of the beamspace CSI. However, all of these estimation schemes [16,17,18] are based on model-driven methods, which rely on idealized mathematical representations and thus suffer significant performance degradation under model mismatches caused by complex and nonideal propagation conditions.

Recently, the approximate message passing (AMP) algorithm [19] has attracted considerable attention as an efficient iterative method for sparse signal reconstruction, especially for high-dimensional channel estimation. Motivated by the remarkable success of deep learning (DL), a variety of DL-aided AMP methods have emerged to optimize beamspace channel estimation performance [20,21,22]. In [20], by unfolding the AMP algorithm into a denoising convolutional neural network, a learning-based AMP (LAMP) network was introduced to estimate the beamspace channel. In [21], a fully convolutional denoising AMP network was employed to learn the channel structure and capture noise characteristics, thereby achieving a higher sum-rate and lower estimation error, particularly under low SNR conditions. In [22], a combined residual network and AMP-based algorithm was considered to improve estimation performance. While these data-driven approaches leverage the powerful feature-learning potential of neural networks for performance enhancement, they typically require large-scale labeled datasets and suffer from limited model interpretability. To this end, a learning-based Gaussian mixture LAMP (GM-LAMP) network was introduced in [23], which exploits the beamspace channel priors to mitigate these drawbacks. By utilizing the derived shrinkage function, the GM-LAMP network can achieve better performance.

However, both AMP-based and AMP-DL algorithms are typically designed under the independent and identically distributed (i.i.d.) property of the measurement matrix. In a number of applications, measurement matrices often exhibit ill-conditioning and depart from i.i.d. characteristics, which significantly restricts the applicability of these methods. In such scenarios, the AMP algorithm may exhibit instability, leading to poor performance or even divergence.

### 1.3. Contributions

To overcome the drawbacks of the existing methods, we develop a model-data hybrid-driven network (MD-HDN) for wideband beamspace channel estimation by combining DL models with channel prior information. The primary contributions of this work are summarized as follows:

- We derive a new shrinkage function for the vector approximate message passing (VAMP) algorithm, where the Gaussian mixture model (GMM) and the expectation-maximization (EM) algorithm are employed to adaptively learn the beamspace channel characteristics, and thus enhance estimation accuracy.
- We develop a deep unfolding architecture by mapping the VAMP algorithm onto a multilayer neural network, which combines model-driven interpretability and data-driven adaptability to optimize wideband beamspace channel estimation.
- We provide extensive simulation results to validate the effectiveness of the proposed MD-HDN scheme, which exhibits significant advantages over state-of-the-art methods in terms of estimation accuracy and robustness.

The remainder of this paper is organized as follows. First, wideband beamspace channel estimation is characterized as a sparse signal recovery problem, especially considering the beam-squint effect. A novel shrinkage function for the VAMP algorithm is then derived and employed in the proposed MD-HDN scheme to enhance estimation accuracy. Subsequently, simulation results are performed to validate the effectiveness and superiority of the MD-HDN scheme compared with existing methods. Finally, conclusions are drawn.

## 2. Channel Model and Problem Formulation

**Figure 1:**
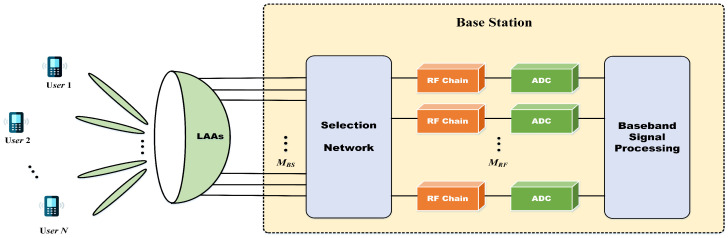
The wideband mmWave Massive MIMO-OFDM system equipped with LAAs.

This section begins by introducing the beamspace channel model for wideband millimeter-wave (mmWave) massive MIMO systems, especially concerned with the beam-squint effect. Building on this model, we formulate wideband beamspace channel estimation as a sparse signal recovery problem. As depicted in Figure 1, the considered uplink time-division duplex (TDD) mmWave massive MIMO-OFDM system comprises the base station (BS) equipped with $M_{\mathrm{RF}}$ RF chains and an $M_{\mathrm{BS}}$-element LAAs to simultaneously communicate with *N* users.

### 2.1. Wideband Beamspace Channel Model

To address the wideband beamspace channel estimation problem, the spatial-domain channel is first established. Specifically, by employing the Saleh–Valenzuela channel model [24], the $M_{\mathrm{BS}}\times 1$ channel vector $h_{k}$ between a given user and the BS at the *k*-th subcarrier (k=1,2,⋯,K) is defined as

(1) $$h_{k}=\sqrt{\frac{M_{\mathrm{BS}}}{L}}\sum_{l=1}^{L}\xi_{l}e^{-j2\pi \tau_{l}f_{k}}a\left(\vartheta_{l,k}\right),$$

where $\xi_{l}$ and $\tau_{l}$ represent the complex gain and the propagation delay of the *l*-th path, while *L* denotes the total number of resolvable paths. Moreover, $\vartheta_{l,k}$ characterizes the spatial direction for the *l*-th path at the *k*-th subcarrier, which is given by

(2) $$\vartheta_{l,k}=\frac{f_{k}}{c}d\sin\theta_{l},$$

where *c* denotes the speed of light, and $\theta_{l}\in \left[-\pi /2,\pi /2\right]$ represents the physical direction of the *l*-th path. Additionally, $f_{k}$ is the frequency of the *k*-th subcarrier, which can be expressed as

(3) $$f_{k}=f_{c}+\frac{f_{s}}{K}\left(k-1-\frac{K-1}{2}\right),$$

where $f_{s}$ and $f_{c}$ are the sampling rate (bandwidth) and carrier frequency, respectively. The antenna spacing *d* is typically set to $d=c/2f_{c}$ [25]. According to (2) and (3), the spatial direction $\vartheta_{l,k}$ is frequency-dependent in wideband mmWave systems. In contrast, for narrowband mmWave systems, $\vartheta_{l,k}$ is frequency-independent due to the condition $f_{s}\ll f_{c}$. Furthermore, for a standard uniform linear array (ULA) with $M_{\mathrm{BS}}$ elements, the array steering vector $a(\vartheta_{l,k})$ in (1) can be defined as

(4) $$a\left(\vartheta_{l,k}\right)=\frac{1}{\sqrt{M_{\mathrm{BS}}}}e^{-j2\pi \vartheta_{l,k}I},$$

where $I={\left[-\frac{M_{\mathrm{BS}}-1}{2},-\frac{M_{\mathrm{BS}}+1}{2},\cdots ,\frac{M_{\mathrm{BS}}-1}{2}\right]}^{T}$ is the set of integers symmetric about zero.

As illustrated in Figure 1, the LAAs are employed in the BS to transform the spatial-domain channel vector $h_{k}$ to the beamspace domain. Specifically, the LAAs fundamentally perform a spatial Discrete Fourier Transform (DFT), characterized by the matrix F. Accordingly, the beamspace channel vector ${\tilde{h}}_{k}$ is given by

(5) $$\begin{array}{cc} {\tilde{h}}_{k}≜Fh_{k} & =F\sqrt{\frac{M_{\mathrm{BS}}}{L}}\sum_{l=1}^{L}\xi_{l}e^{-j2\pi \tau_{l}f_{k}}a\left(\vartheta_{l,k}\right)\\ & =\sqrt{\frac{M_{\mathrm{BS}}}{L}}\sum_{l=1}^{L}\xi_{l}e^{-j2\pi \tau_{l}f_{k}}{\tilde{b}}_{l,k}, \end{array}$$

where F is composed of $M_{\mathrm{BS}}$ orthogonal array steering vectors and is formulated as

(6) $$F={\left[a\left({\bar{\vartheta }}_{1}\right),a\left({\bar{\vartheta }}_{2}\right),\cdots ,a\left({\bar{\vartheta }}_{M_{\mathrm{BS}}}\right)\right]}^{H},$$

where ${\bar{\vartheta }}_{i}=\frac{1}{M_{\mathrm{BS}}}\left(i-\frac{M_{\mathrm{BS}}+1}{2}\right)$, for $i=1,2,\ldots ,M_{\mathrm{BS}}$ denotes the discrete spatial direction associated with the LAAs. Furthermore, the beamspace component of the *l*-th path at the *k*-th subcarrier is represented by the vector ${\tilde{b}}_{l,k}$, which is given by

(7) $$\begin{array}{cc} {\tilde{b}}_{l,k} & =Fa\left(\vartheta_{l,k}\right)\\ & ={\left[\Theta \left(\vartheta_{l,k}-{\bar{\vartheta }}_{1}\right),\Theta \left(\vartheta_{l,k}-{\bar{\vartheta }}_{2}\right),\cdots ,\Theta \left(\vartheta_{l,k}-{\bar{\vartheta }}_{M_{\mathrm{BS}}}\right)\right]}^{T}, \end{array}$$

where $\Theta \left(x\right)≜\frac{\sin M_{\mathrm{BS}}\pi x}{\sin\pi x}$ represents the Dirichlet sinc function.

Due to the energy concentration of the Θ(x) function [7], the energy of ${\tilde{b}}_{l,k}$ is concentrated within only a limited number of beam directions. Furthermore, the limited scattering effect in mmWave systems results in a small number of propagation paths [4]. Therefore, the beamspace channel vector ${\tilde{h}}_{k}$ exhibits a distinct sparse structure [17].

### 2.2. Problem Formulation

In TDD-based communication systems, the channel is considered to be unchanged during the pilot transmission phase for channel estimation. To facilitate independent estimation for multiple users, an orthogonal pilot scheme is utilized in the uplink. Due to the inherent channel reciprocity in TDD systems, the downlink CSI can be acquired from the uplink estimates without additional overhead. Accordingly, the $M_{RF}\times 1$ received signal $y_{k,q}$ at the *k*-th subcarrier and instant *q* is given by

(8) $$y_{k,q}=D_{q}{\tilde{h}}_{k}p_{k,q}+D_{q}n_{k,q},\;k=1,2,\cdots ,K,$$

where $D_{q}$ is the $M_{RF}\times M_{BS}$ hybrid combining matrix used for beam selection, $p_{k,q}$ is the pilot symbol, and $n_{k,q}∼\mathrm{CN}\left(0,\sigma^{2}I_{M_{BS}}\right)$ is the $M_{BS}\times 1$ complex Gaussian noise vector with variance $\sigma^{2}$. Following the transmission of *Q* pilot symbols with $p_{k,q}=1$, the $QM_{RF}\times 1$ overall received signal ${\bar{y}}_{k}$ can be written as

(9) $${\bar{y}}_{k}=\left[\begin{array}{c} y_{k,1}\\ y_{k,2}\\ \vdots \\ y_{k,Q} \end{array}\right]=\bar{D}{\tilde{h}}_{k}+{\bar{n}}_{k},\;k=1,2,\cdots ,K,$$

where $\bar{D}={\left[D_{1}^{T},D_{2}^{T},\cdots ,D_{Q}^{T}\right]}^{T}$ represents the $QM_{RF}\times M_{BS}$ hybrid matrix, whose elements are drawn i.i.d. from the discrete set $\left\{-\frac{1}{\sqrt{QM_{RF}}},+\frac{1}{\sqrt{QM_{RF}}}\right\}$ with uniform probability. Additionally, ${\bar{n}}_{k}$ denotes the effective noise vector.

According to (9), the wideband beamspace channel vector ${\tilde{h}}_{k}$ is recoverable from ${\bar{y}}_{k}$ and $\bar{D}$. Owing to the sparse scattering nature of mmWave propagation, only a limited number of multipath components exhibit significant gains, resulting in an approximately sparse beamspace channel. Furthermore, since the number of RF chains is typically much smaller than the number of BS antennas, the wideband channel estimation task can be characterized as an underdetermined sparse recovery problem. Therefore, the channel estimation in (9) is essentially a compressive sensing (CS) problem, which can be addressed using established CS-based algorithms.

(10) $$\min{∥{\tilde{h}}_{k}∥}_{0},\;s.t.{∥{\bar{y}}_{k}-\bar{D}{\tilde{h}}_{k}∥}_{2}\leq \eta ,$$

where η>0 represents the error tolerance parameter, and ${∥{\tilde{h}}_{k}∥}_{0}$ denotes the sparsity level (i.e., the number of non-zero entries) of the channel vector. Since $\ell_{0}$-norm minimization is NP-hard owing to its non-convexity, it is commonly approximated via $\ell_{1}$-norm relaxation. Although conventional CS algorithms, including OMP [16], SSD [17], and AMP [19], have been proposed to solve this problem, these algorithms often fail to yield satisfactory estimation performance. In particular, their performance degrades significantly when the measurement matrix is ill-conditioned or the i.i.d. assumption is no longer valid.

## 3. Model-Data Hybrid-Driven Channel Estimation Scheme

In this section, the VAMP algorithm is first introduced as the foundation for estimating the beamspace channel. Then, to further improve estimation performance, we derive a novel shrinkage function based on the Gaussian mixture characteristics of the beamspace channel elements. Building on this derived function, we further develop an MD-HDN scheme to achieve enhanced estimation accuracy. Finally, we provide a comprehensive computational complexity analysis to compare the proposed scheme with existing algorithms.

### 3.1. VAMP-Based Wideband Beamspace Channel Estimation

To address the challenge of wideband beamspace channel estimation, various CS-based methods have been developed by solving the problem formulated in (10). While the AMP algorithm is highly efficient for high-dimensional sparse signal reconstruction, its efficacy is strictly limited to the i.i.d. property of the measurement matrix. Otherwise, AMP is prone to instability, potentially leading to severe performance deterioration or even divergence. To overcome these limitations, the VAMP algorithm was proposed as a robust extension designed to handle more general measurement matrices [26]. Specifically, by employing the economy-size singular value decomposition (SVD), the measurement matrix $A\in R^{M\times N}$ in the VAMP algorithm can be decomposed as

(11) $$A=U\mathrm{Diag}\left(s\right)V^{T},$$

where $s\in R^{R}$ is the vector of its positive singular values, and R=rank(A)≤min(M,N) denotes the rank of A. The matrix V is formed by the first *R* columns of an N×N orthogonal matrix generated uniformly. A notable advantage of VAMP is its robustness to arbitrary singular values s and the orthogonal matrix U, provided that both *M* and *N* are sufficiently large. Given the noisy linear model in (9), the channel vector ${\tilde{h}}_{k}\left(k=1,2,\cdots ,K\right)$ can be estimated by reformulating (9) as

(12) $$\bar{Y}=\bar{D}\tilde{H}+\bar{N},$$

where $\bar{N}=\left[{\bar{n}}_{1},{\bar{n}}_{2},\ldots ,{\bar{n}}_{K}\right]$, $\bar{Y}=\left[{\bar{y}}_{1},{\bar{y}}_{2},\ldots ,{\bar{y}}_{K}\right]$, and $\tilde{H}=\left[{\tilde{h}}_{1},{\tilde{h}}_{2},\ldots ,{\tilde{h}}_{K}\right]$.

Motivated by the strong performance of VAMP against ill-conditioned and structured measurement matrices, we design a VAMP-based estimator to facilitate beamspace channel estimation. The flow of the proposed estimator, as summarized in Algorithm 1, alternates between a linear minimum mean square error (LMMSE) stage and a nonlinear shrinkage (denoising) stage. Each stage executes a consistent set of four computational steps: state estimation, divergence calculation, Onsager correction, and variance update. The primary distinction between the two stages lies in the type of estimator employed in each.

**Algorithm 1:** VAMP-BasedWideband Beamspace Channel Estimation

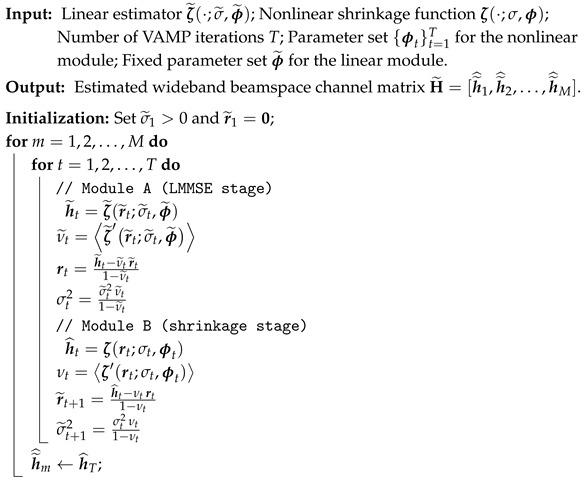



In the first stage, the estimator $\tilde{\zeta }\left({\tilde{r}}_{t};{\tilde{\sigma }}_{t},\tilde{\phi }\right)$ is given by

(13) $$\tilde{\zeta }\left({\tilde{r}}_{t};{\tilde{\sigma }}_{t},\tilde{\phi }\right)≜V{\left(Diag{\left(s\right)}^{2}+\frac{\sigma_{w}^{2}}{{\tilde{\sigma }}_{t}^{2}}I_{R}\right)}^{-1}\left(Diag\left(s\right)U^{T}{\bar{y}}_{m}+\frac{\sigma_{w}^{2}}{{\tilde{\sigma }}_{t}^{2}}V^{T}{\tilde{r}}_{t}\right),$$

which depends on the measurement vector ${\bar{y}}_{k}$ and the parameter set $\tilde{\phi }$, defined as

(14) $$\tilde{\phi }≜\left\{U,s,V,\sigma_{w}\right\}.$$

Here, U and V denote the unitary matrices obtained from the SVD of the measurement matrix, s is the singular values, and $\sigma_{w}^{2}$ is the measurement noise variance. In the second stage, the estimator $\zeta (r_{t};\sigma_{t},\phi_{t})$ performs nonlinear shrinkage using several shrinkage functions, such as the scaled soft-threshold, piecewise-linear, exponential, spline, and Bernoulli–Gaussian functions [27]. Notably, ${\tilde{\sigma }}_{t}^{2}$ and $\sigma_{t}^{2}$ represent the effective noise variance of the pseudo-prior and pseudo-observation at different stages of the iteration process, respectively. Specifically, ${\tilde{\sigma }}_{t}^{2}$ characterises the inherent uncertainty of the prior information, while $\sigma_{t}^{2}$ captures the residual error from the measurement process.

The divergence of the estimators $\tilde{\zeta }\left({\tilde{r}}_{t};{\tilde{\sigma }}_{t},\tilde{\phi }\right)$ and $\zeta (r_{t};\sigma_{t},\phi_{t})$, defined as the arithmetic mean of their respective Jacobian diagonal entries, is calculated in Algorithm 1. Specifically, for the LMMSE stage, the Jacobian associated with $\tilde{\zeta }\left({\tilde{r}}_{t};{\tilde{\sigma }}_{t},\tilde{\phi }\right)$ is given by

(15) $$\frac{\sigma_{w}^{2}}{{\tilde{\sigma }}_{t}^{2}}V{\left(Diag{\left(s\right)}^{2}+\frac{\sigma_{w}^{2}}{{\tilde{\sigma }}_{t}^{2}}I_{R}\right)}^{-1}V^{T},$$

and then the average of its diagonal is

(16) $$\langle {\tilde{\zeta }}^{\prime }\left({\tilde{r}}_{t};{\tilde{\sigma }}_{t},\tilde{\phi }\right)\rangle ≜\frac{1}{N}\sum_{i=1}^{R}\frac{1}{\frac{s_{i}^{2}{\tilde{\sigma }}_{t}^{2}}{\sigma_{w}^{2}}+1}.$$

In the shrinkage stage, the divergence of the estimator $\zeta (r_{t};\sigma_{t},\phi_{t})$ is computed as

(17) $$\langle \zeta^{\prime }\left(r_{t};\sigma_{t},\phi_{t}\right)\rangle ≜\frac{1}{N}\sum_{j=1}^{N}\frac{\partial {\left[\zeta (r_{t};\sigma_{t},\phi_{t})\right]}_{j}}{\partial r_{j}}.$$

**Figure 2:**
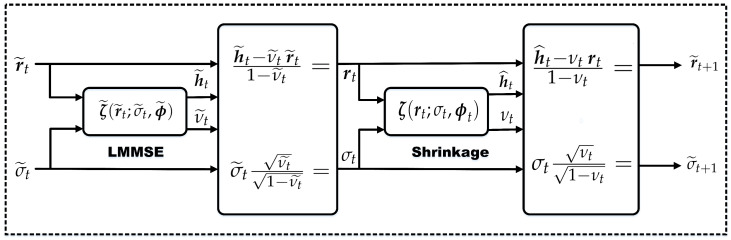
The structure of VAMP-based wideband beamspace channel estimation.

As illustrated in Figure 2, the VAMP algorithm alternates between LMMSE estimation and nonlinear shrinkage, which achieves high accuracy in high-dimensional sparse signal recovery. In each iteration, the residual vectors $r_{t}$ and ${\tilde{r}}_{t}$ are adjusted by the Onsager correction terms $-{\tilde{\nu }}_{t}\;{\tilde{r}}_{t}$ and $-\nu_{t}\;r_{t}$. These terms correspond to the divergences of the estimators $\tilde{\zeta }\left({\tilde{r}}_{t};{\tilde{\sigma }}_{t},\tilde{\phi }\right)$ and $\zeta (r_{t};\sigma_{t},\phi_{t})$, respectively. In VAMP-based channel estimation, the shrinkage function plays a critical role by integrating prior channel information into the iterative optimization of observed measurements. This operation simultaneously reduces error propagation and accelerates convergence, thus achieving high-precision channel reconstruction. The performance of the shrinkage function critically depends on the alignment between the prior model and the actual channel characteristics. When the alignment is poor, estimation accuracy is fundamentally limited. Conventional shrinkage functions, such as soft-thresholding, piecewise-linear, and Bernoulli–Gaussian models are generally designed for generic sparse signal recovery and do not exploit the unique structure of wideband beamspace channels. In particular, they cannot capture the structured sparsity and nonstationary statistics induced by beam squint and multipath propagation. Moreover, these generic shrinkage functions rely heavily on manually tuned thresholds or hyperparameters, limiting their ability to adapt dynamically to time-varying channel conditions.

### 3.2. Proposed EM-GMM Shrinkage Function

To address these limitations, an EM-GMM-based shrinkage function is introduced for beamspace channel estimation. Specifically, the beamspace channel distribution is represented by leveraging the GMM, in which each Gaussian component is associated with a distinct angular cluster of the channel. Model parameters are then directly estimated from the observed data via the EM algorithm, without the empirical parameters. By integrating the EM algorithm into the VAMP iterations, the prior parameters are adaptively learned from the beamspace channel, which enables more accurate recovery of the sparse channel during the iterative process.

Let $\bar{h}$ denote the beamspace channel vector, where $\bar{h}$ is an arbitrary element of $\bar{h}$. Then, the prior distribution of $\bar{h}$ is given by a mixture with $N_{c}$-component complex Gaussian components, which can be represented as

(18) $$p\left(\bar{h};\theta \right)=\sum_{k=0}^{N_{c}-1}p_{k}\;\mathrm{CN}\left(\bar{h};\mu_{k},\sigma_{k}^{2}\right),$$

where $p_{k}$ denotes the mixing weight of the *k*-th Gaussian component, $\mu_{k}$ and $\sigma_{k}^{2}$ are its mean and variance, respectively. The set of prior parameters is denoted by $\theta ={\left\{p_{k},\mu_{k},\sigma_{k}^{2}\right\}}_{k=0}^{N_{c}-1}$. The probability density function (PDF) of an arbitrary channel element $\bar{h}$ under the *k*-th Gaussian component is then given by

(19) $$\mathrm{CN}\left(\bar{h};\mu_{k},\sigma_{k}^{2}\right)=\frac{1}{\pi \sigma_{k}^{2}}\exp\left(-\frac{{\left|\bar{h}-\mu_{k}\right|}^{2}}{\sigma_{k}^{2}}\right).$$

According to (18) and (19), the complex GMM can flexibly characterize both the sparsity and distribution of the beamspace channel. Each mixture component corresponds to a distinct scattering cluster or a specific power level, which enables the prior model to capture non-uniform energy distributions in the spatial direction.

Then, based on the Bayesian MMSE estimation, we derive the shrinkage function $\zeta_{\mathrm{GMM}}$ under the complex GMM, expressed as

(20) $$\zeta_{\mathrm{GMM}}\left(r;\sigma^{2},\theta \right)=E\left[\bar{h}|r;\sigma^{2},\theta \right]=\frac{\int \bar{h}\;p\left(r|\bar{h};\sigma^{2}\right)\;p\left(\bar{h};\theta \right)\;d\bar{h}}{\int p\left(r|\bar{h};\sigma^{2}\right)\;p\left(\bar{h};\theta \right)\;d\bar{h}}.$$

where the input *r* denotes the effective observation generated by the VAMP algorithm, as defined in [27]

(21) $$r=\bar{h}+n,$$

where $n∼\mathrm{CN}(0,\sigma^{2})$ denotes the additive Gaussian noise. Accordingly, the likelihood of the observation *r* given the channel $\bar{h}$ is

(22) $$p\left(r|\bar{h};\sigma^{2}\right)=\mathrm{CN}\left(r;\bar{h},\sigma^{2}\right).$$

Combining the likelihood $p(r|\bar{h};\sigma^{2})$ with the prior $p(\bar{h};\theta )$, we derive the MMSE estimate of $\bar{h}$, which serves as the shrinkage function in the VAMP algorithm:

(23) $$\begin{array}{cc} p\left(r|\bar{h};\sigma^{2}\right)p\left(\bar{h};\theta \right) & =\mathrm{CN}\left(r;\bar{h},\sigma^{2}\right)\sum_{k=0}^{N_{c}-1}p_{k}\;\mathrm{CN}\left(\bar{h};\mu_{k},\sigma_{k}^{2}\right)\\ \; & =\sum_{k=0}^{N_{c}-1}p_{k}CN\left(r;\bar{h},\sigma^{2}\right)CN\left(\bar{h};\mu_{k},\sigma_{k}^{2}\right)\\ \; & =\sum_{k=0}^{N_{c}-1}p_{k}CN\left(r;\mu_{k},\sigma^{2}+\sigma_{k}^{2}\right)CN\left(\bar{h};{\tilde{\mu }}_{k}\left(r\right),{\tilde{\sigma }}_{k}^{2}\right), \end{array}$$

where ${\tilde{\mu }}_{k}\left(r\right)=\frac{\sigma_{k}^{2}\;r+\sigma^{2}\;\mu_{k}}{\sigma^{2}+\sigma_{k}^{2}}$ and ${\tilde{\sigma }}_{k}^{2}=\frac{\sigma^{2}\sigma_{k}^{2}}{\sigma^{2}+\sigma_{k}^{2}}$.

Finally, by substituting (23) in (20), the shrinkage function $\zeta_{\mathrm{GMM}}$ based on the complex GMM can be explicitly written as

(24) $$\zeta_{\mathrm{GMM}}\left(r;\sigma^{2},\theta \right)=\frac{\sum_{k=0}^{N_{c}-1}p_{k}\;{\tilde{\mu }}_{k}\left(r\right)\;\mathrm{CN}\;\left(r;\mu_{k},\sigma^{2}+\sigma_{k}^{2}\right)}{\sum_{k=0}^{N_{c}-1}p_{k}\;\mathrm{CN}\;\left(r;\mu_{k},\sigma^{2}+\sigma_{k}^{2}\right)}.$$

Equation (25) shows that $\zeta_{\mathrm{GMM}}\left(r;\sigma^{2},\theta \right)$ depends on the prior parameters θ, which are generally unknown in practice and may vary significantly with the channel environment. Therefore, the EM algorithm is integrated into the derived shrinkage function to adaptively estimate θ from the current set of observations ${\left\{r_{m}\right\}}_{m=1}^{M}$. Specifically, in the expectation step (E-step), the posterior responsibility of the *k*-th Gaussian component for the observation $r_{m}$ is given by

(25) $$\omega_{k}\left(r_{m}\right)=\frac{p_{k}^{\left(t\right)}\;\mathrm{CN}\left(r_{m};\mu_{k}^{\left(t\right)},\sigma_{k}^{2(t)}+\sigma^{2}\right)}{\sum_{j=0}^{N_{c}-1}p_{j}^{\left(t\right)}\;\mathrm{CN}\left(r_{m};\mu_{j}^{\left(t\right)},\sigma_{j}^{2(t)}+\sigma^{2}\right)}.$$

In the maximum step (M-step), the parameters are updated using the posterior probability as

(26) $$\begin{array}{cc} p_{k}^{\left(t+1\right)} & =\frac{1}{M}\sum_{m=1}^{M}\omega_{k}\left(r_{m}\right), \end{array}$$

(27) $$\begin{array}{cc} \mu_{k}^{\left(t+1\right)} & =\frac{\sum_{m=1}^{M}\omega_{k}\left(r_{m}\right)\;{\tilde{\mu }}_{k}^{\left(t\right)}\left(r_{m}\right)}{\sum_{m=1}^{M}\omega_{k}\left(r_{m}\right)}, \end{array}$$

(28) $$\begin{array}{cc} \sigma_{k}^{2(t+1)} & =\frac{\sum_{m=1}^{M}\omega_{k}\left(r_{m}\right)\left(|{\tilde{\mu }}_{k}^{\left(t\right)}\left(r_{m}\right)-\mu_{k}^{\left(t+1\right)}{|}^{2}+{\tilde{\sigma }}_{k}^{2(t)}\right)}{\sum_{m=1}^{M}\omega_{k}\left(r_{m}\right)}, \end{array}$$

where ${\tilde{\mu }}_{k}^{\left(t\right)}\left(r_{m}\right)$ and ${\tilde{\sigma }}_{k}^{2(t)}$ are computed with $\mu_{k}^{\left(t\right)}$ and $\sigma_{k}^{2(t)}$ in (24).

After updating the parameters, the proposed shrinkage function $\zeta_{\mathrm{EM}-\mathrm{GMM}}\left(r;\sigma^{2},\theta \right)$ can be represented as

(29) $$\zeta_{\mathrm{EM}-\mathrm{GMM}}\left(r;\sigma^{2},\theta \right)=\frac{\sum_{k=0}^{N_{c}-1}{\hat{p}}_{k}\;{\tilde{\mu }}_{k}\left(r\right)\;\mathrm{CN}\;\left(r;{\hat{\mu }}_{k},\sigma^{2}+{\hat{\sigma }}_{k}^{2}\right)}{\sum_{k=0}^{N_{c}-1}{\hat{p}}_{k}\;\mathrm{CN}\;\left(r;{\hat{\mu }}_{k},\sigma^{2}+{\hat{\sigma }}_{k}^{2}\right)},$$

where $\left\{{\hat{p}}_{k},{\hat{\mu }}_{k},{\hat{\sigma }}_{k}^{2}\right\}$ are the EM-updated parameters. In contrast to the conventional shrinkage function employed in the standard VAMP algorithm, the proposed shrinkage function can adaptively optimize its parameters at each iteration, thereby enhancing the accuracy and robustness of channel estimation.

### 3.3. The Proposed MD-HDN Estimation Scheme

While the VAMP algorithm achieves significant gains in estimation accuracy by leveraging the derived shrinkage function, it may suffer performance degradation in practical scenarios characterized by model mismatches, hardware impairments, or time-varying propagation conditions. In these cases, the assumed statistical prior may become invalid, thereby degrading the estimation accuracy. In contrast, data-driven methods can learn statistical characteristics of unknown channels from large-scale datasets. However, they typically lack clear physical interpretability. To address these limitations, we propose the MD-HDN scheme by unfolding the iterative VAMP algorithm into a neural network architecture, which effectively integrates model-based physical knowledge with adaptive data-driven learning. Notably, the proposed scheme retains the VAMP framework but replaces its conventional shrinkage function with the derived function.

**Figure 3:**
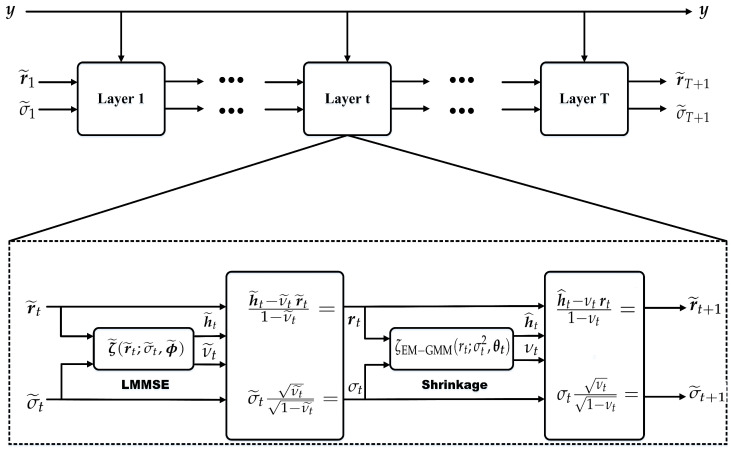
The structure of the proposed MD-HDN scheme.

As illustrated in Figure 3, the proposed MD-HDN scheme includes *T* homogeneous layers with similar input-output structures and dimensions. At the *t*-th layer, given the measurements y, the channel estimate ${\hat{\tilde{h}}}_{t}$ is computed as

(30) $$r_{t}=\frac{{\tilde{h}}_{t}-{\tilde{\nu }}_{t}\;{\tilde{r}}_{t}}{1-{\tilde{\nu }}_{t}}$$

(31) $${\hat{h}}_{t}=\zeta_{\mathrm{EM}-\mathrm{GMM}}\left(r_{t};\sigma_{t}^{2},\theta_{t}\right),$$

where $r_{t}$ is the effective observation fed into the *t*-th layer, $\sigma_{t}^{2}$ is the residual noise variance estimated via the VAMP Onsager correction, $\theta_{t}$ represents the layer-specific GMM parameters updated via the EM algorithm, and $\zeta_{\mathrm{EM}-\mathrm{GMM}}\left(\cdot \right)$ is the derived MMSE-optimal shrinkage function based on the complex GMM prior.

The MD-HDN follows the standard deep unfolding paradigm, including an offline training stage and an online inference stage. In training, the network is optimized over a large dataset of labeled channel realizations by minimizing the loss function for all trainable variables $\Theta_{t}={\left\{\tilde{\theta_{k}},\;\theta_{k}\right\}}_{k=1}^{t}$. Subsequently, during online estimation, the trained network maps the observed measurements y directly to the channel estimate ${\hat{\tilde{h}}}_{t}$.

Offline training of the MD-HDN follows a supervised paradigm based on ${\left\{y^{d},{\tilde{h}}^{d}\right\}}_{d=1}^{D}$, where each measurement vector $y^{d}$ is associated with its true beamspace channel ${\tilde{h}}^{d}$. To alleviate overfitting and enhance training stability, we adopt the layer-by-layer training strategy proposed in [27]. Specifically, the overall optimization is decomposed into *T* sequential sub-processes. During the *t*-th sub-process (t=1,…,T), the parameter set $\Theta_{t}={\left\{\tilde{\theta_{k}},\;\theta_{k}\right\}}_{k=1}^{t}$ is updated by minimizing two complementary loss functions, which are specifically designed to supervise the linear MMSE update and the nonlinear EM-GMM shrinkage, respectively:

(32) $$L_{t}^{\mathrm{linear}}\left(\Theta_{t}\right)=\frac{1}{D}\sum_{d=1}^{D}{∥r_{t}^{d}\left(y^{d},\Theta_{t}\right)-{\tilde{h}}^{d}∥}_{2}^{2},$$

(33) $$L_{t}^{\mathrm{nonlinear}}\left(\Theta_{t}\right)=\frac{1}{D}\sum_{d=1}^{D}{∥{\hat{\tilde{h}}}_{t}^{d}\left(y^{d},\Theta_{t}\right)-{\tilde{h}}^{d}∥}_{2}^{2},$$

where $h_{t}^{d}$ and $r_{t}^{d}$ denote the outputs of the linear and nonlinear operations defined in (32) and (33), respectively. To balance identifiability and convergence stability, a hybrid optimization strategy, combining individual and joint optimization, is employed during the whole training procedure. First, the linear coefficients of the *t*-th layer are optimized while all other parameters are held fixed. Second, the nonlinear EM-GMM shrinkage parameters of the same layer are updated independently. Finally, a joint optimization is performed over the entire parameter set $\Theta_{t}={\left\{\tilde{\theta_{k}},\;\theta_{k}\right\}}_{k=1}^{t}$, which encompasses all trainable parameters from layers 1 through *t*. According to the aforementioned three-step procedure, the trained MD-HDN network can achieve efficient fine-tuning at each layer, and thus avoid locally suboptimal solutions caused by overfitting.

In the online phase, the pre-trained MD-HDN is utilized to enable real-time wideband beamspace channel estimation. Specifically, the received measurement vector y is fed into the network, which directly outputs the corresponding channel estimate $\hat{\tilde{h}}$. To objectively benchmark its accuracy, we adopt the normalized mean square error (NMSE) as the evaluation metric, defined as

(34) $$\mathrm{NMSE}=\frac{E\left\{\sum_{k=1}^{K}{∥{\hat{\tilde{h}}}_{k}-{\tilde{h}}_{k}∥}_{2}^{2}\right\}}{E\left\{\sum_{k=1}^{K}{∥{\tilde{h}}_{k}∥}_{2}^{2}\right\}}.$$

### 3.4. Computational Complexity Analysis

While deep unfolding methods generally increase model expressiveness at the cost of computation, the proposed MD-HDN maintains a modest complexity profile. For AMP-based methods, namely, LAMP [20] and GM-LAMP [23], each iteration involves O(MN) operations, resulting in a total complexity of O(TMN) over *T* layers. Similarly, the VAMP-based approaches, including LVAMP [27] and the proposed MD-HDN, follow a similar computational pattern and share a complexity of O(TMN), due to their iterative structure involving matrix-vector multiplications. In contrast, the OMP algorithm has a complexity of $O\left(SMN\right)+O\left(S^{3}M\right)$, where *S* denotes the sparsity level of the beamspace channel vector.

Although the proposed MD-HDN shares the same asymptotic complexity O(TMN) as other deep unfolding methods, its per-iteration EM-GMM adaptation introduces a modest constant-factor overhead due to responsibility computation and online updates of Gaussian component parameters. To quantify this trade-off, we provide empirical inference times, training time and memory measured on an NVIDIA RTX 4090 GPU using PyTorch (version 2.10) under a 256×1 ULA configuration, where beamspace dimension M=256, pilot N=64 and T=10. As summarized in Table 1, MD-HDN requires approximately 12.5 ms per channel sample about 1.8× slower than LAMP, yet still well within the coherence time of typical mmWave channels (10–100 ms). Given its consistent NMSE gains of over 1.5 dB across SNR regimes and array geometries, this additional computational cost is justified by the significantly improved estimation accuracy.

## 4. Simulation Results and Analysis

To validate its efficacy, the proposed MD-HDN method is evaluated against several state-of-the-art channel estimation methods, including conventional iterative methods (OMP algorithm [16], AMP algorithm [19], VAMP algorithm [26]) and deep unfolding networks (LAMP network [20], GM-LAMP network [23], LVAMP network [27], LDGEC network [28], AMP-SBL unfolding [29], and DLISTA [30]). Moreover, extensive numerical results are presented based on the open-source DeepMIMO dataset [31] and the Saleh–Valenzuela model.

### 4.1. Simulation Setup

Our evaluation is based on a wideband mmWave massive MIMO-OFDM system, in which the base station (BS) is equipped with $M_{\mathrm{BS}}=256$ antenna elements and $M_{\mathrm{RF}}=16$ RF chains to serve N=16 single-antenna users. The carrier frequency and bandwidth are set to $f_{c}=28$ GHz and $f_{s}=4$ GHz, respectively, with K=512 OFDM subcarriers. For each user, we collect M=128 measurements, and the uplink SNR is defined as $1/\sigma_{n}^{2}$.

To support robust learning and ensure generalization, we generate a synthetic dataset based on the Saleh–Valenzuela channel model, whose geometric and statistical parameters are listed in Table 2. The dataset consists of 80,000 samples for training, 2000 for validation, and 2000 for testing.

The DeepMIMO dataset is generated using high-fidelity ray-tracing simulations that accurately model mmWave channel characteristics under realistic environmental conditions, such as 3D building geometry and carrier frequency. As detailed in Table 3, the setup comprises 3 active base stations, with mobile users restricted to rows R1000–R1300. To ensure robustness and prevent overfitting, the dataset is split into 50,000 samples for end-to-end training, 2000 validation samples for model selection and early stopping, and 2000 test samples for unbiased performance evaluation. After generating channel realizations from both the DeepMIMO dataset and the Saleh–Valenzuela model, we compute the corresponding beamspace channels and measurement vectors to enable performance evaluation.

Both the LVAMP and the proposed MD-HDN networks are unrolled into T=8 layers. The dimensionality of each layer matches that of the beamspace channel and the corresponding measurement vector. We employ a layer-wise pretraining strategy followed by end-to-end fine-tuning to optimize all trainable parameters. Pretraining employs the Adam optimizer with a fixed mini-batch size of 128. During layer-wise pretraining, the learning rate is held constant at $1\times 10^{-3}$. During joint fine-tuning, the learning rate is reduced sequentially from $5\times 10^{-4}$ to $1\times 10^{-4}$ and finally to $1\times 10^{-5}$, whenever the validation loss plateaus for 5 consecutive epochs. To mitigate overfitting, we employ early stopping triggered by the validation NMSE and weight decay with a coefficient of $1\times 10^{-4}$ during joint training. Dropout is not used, as the unrolled iterative architecture inherently offers regularization. Upon convergence, all models are evaluated on the held-out test sets of both datasets.

In the MD-HDN network, the EM-GMM shrinkage function employs $N_{c}=4$ components. Consequently, the nonlinear parameter vector $\theta^{\left(t\right)}$ at layer *t* contains 12 elements, including four mixing probabilities, four means, and four variances. To exploit the sparsity of wideband beamspace channels, we initialize $\theta_{0}=\left\{0.15,0.15,0.15,0.15,0,0,0,0,0,0,0,0\right\}$. In contrast, the LAMP network uses a scalar soft-thresholding parameter initialized to $\lambda_{0}=1$. For comparison, the OMP-based estimator assumes a known sparsity level of S=24 non-zero elements in the beamspace channel vector. The AMP baseline is executed for T=10 iterations with a fixed empirical shrinkage parameter $\lambda_{t}=1.14$ for all *t*.

### 4.2. Simulation Results on the Saleh–Valenzuela Channel Model

In this subsection, we evaluated the proposed EM-GMM shrinkage function and MD-HDN network under the widely adopted Saleh–Valenzuela channel model. Specifically, we compare four VAMP-based shrinkage strategies, including hard thresholding [27], soft-threshold [27], fixed-parameter GMM (Bernoulli–Gaussian) [27], and the proposed EM-GMM. Furthermore, we compare the proposed MD-HDN network with the existing schemes for wideband beamspace channel estimation, such as conventional CS methods and DL-based networks.

**Figure 4:**
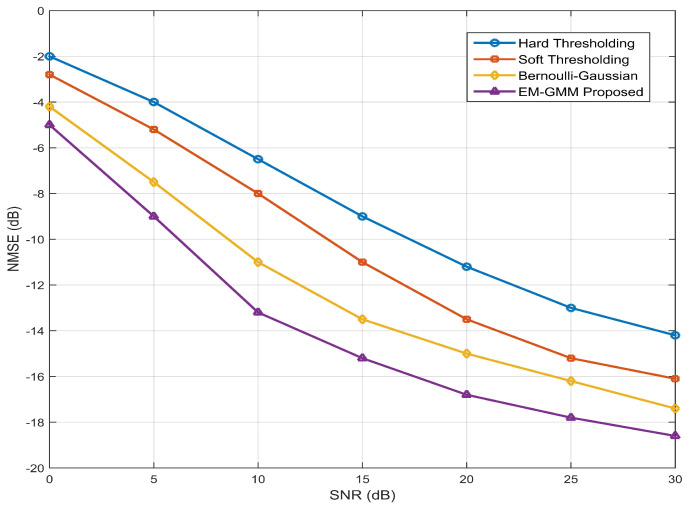
NMSE performance versus SNR for different shrinkage functions in VAMP-based beamspace channel estimation using a 256×1 ULA.

Figure 4 compares the NMSE performance of four shrinkage functions in VAMP-based beamspace channel estimation using a 256×1 ULA. As expected in noise-limited regimes, all methods exhibit decreasing NMSE with increasing SNR. At low SNR (0–10 dB), soft-thresholding slightly outperforms hard-thresholding owing to its continuous shrinkage, which avoids coefficient discontinuities and reduces estimation bias. In the mid-SNR regime (10–20 dB), the Bernoulli–Gaussian approach outperforms thresholding-based methods by leveraging a structured prior that models the sparse statistics of the channel. However, this gain is limited by the assumption of a single Gaussian component, which cannot represent the inherent multi-cluster structure of the beamspace channel. Consequently, the Bernoulli–Gaussian model exhibits unsatisfactory performance at high SNR, where residual bias due to prior-model mismatch dominates the error floor. In contrast, the proposed EM-GMM shrinkage function adapts a multi-component GMM through the EM algorithm updates within each VAMP iteration. This enables effective modeling of the channel statistics, thereby mitigating bias and suppressing noise arising from prior-model mismatch. Hence, the EM-GMM achieves the lowest NMSE across the entire SNR range, confirming the superiority of the proposed shrinkage function.

**Figure 5:**
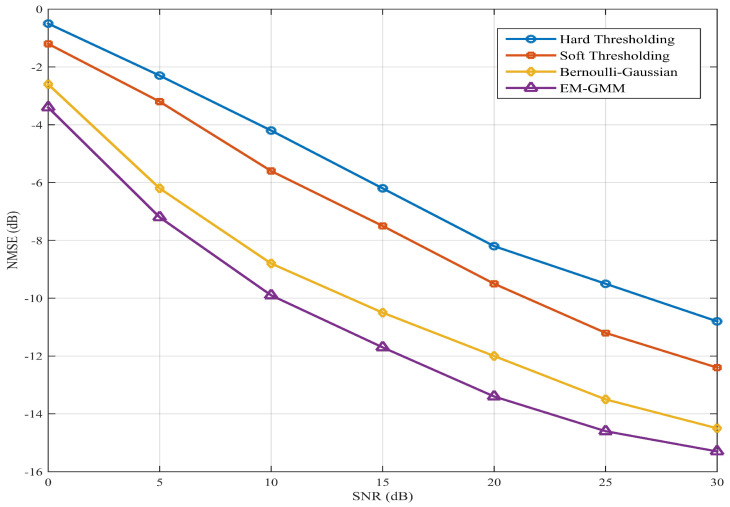
NMSE performance versus SNR for four shrinkage functions in VAMP-based beamspace channel estimation using a 16×16 UPA.

Figure 5 further evaluates the NMSE performance of four shrinkage functions in VAMP-based beamspace channel estimation using a 16×16 UPA. Conventional threshold-based shrinkage functions still yield unsatisfactory estimation performance. The Bernoulli–Gaussian method further improves performance by channel prior information, achieving lower NMSE than both thresholding-based approaches. However, the Bernoulli–Gaussian method exhibits performance saturation beyond 20 dB SNR, as it cannot adequately model the beamspace channel characteristics. In contrast, the proposed shrinkage function leverages channel prior information and an adaptive statistical learning strategy to maintain high estimation accuracy across the entire SNR range. At 30 dB SNR, the proposed shrinkage function outperforms the best Bernoulli–Gaussian method by more than 3 dB, which confirms the superiority of the proposed EM-GMM shrinkage function.

**Figure 6:**
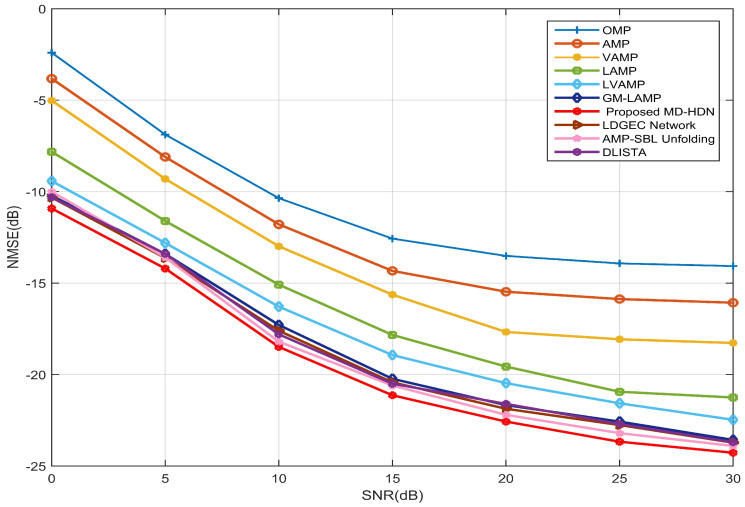
NMSE performance comparison between the proposed MD-HDN network and different algorithms with the ULA configuration under the Saleh–Valenzuela channel model.

In Figure 6, we compare the NMSE performance of the various schemes versus SNR for a 256×1 ULA configuration. The proposed MD-HDN scheme achieves superior estimation performance across the entire SNR range. Specifically, by leveraging large-scale channel data to learn adaptive inference rules, DL-based networks (LAMP, LVAMP, GM-LAMP, LDGEC, AMP-SBL, DLISTA, MD-HDN) significantly outperform model-based methods (OMP, AMP, VAMP), which highlights the benefit of data-driven approaches.

Among these data-driven approaches, the methods based on channel prior information, such as GM-LAMP, AMP-SBL, and MD-HDN, exhibit higher estimation accuracy. In particular, the proposed MD-HDN outperforms LAMP and LVAMP by replacing scalar or fixed shrinkage functions with a learnable Gaussian mixture prior. Moreover, the proposed MD-HDN outperforms both GM-LAMP and AMP-SBL, as its shrinkage function adaptively optimizes its parameters at each iteration to improve estimation accuracy and robustness.

**Figure 7:**
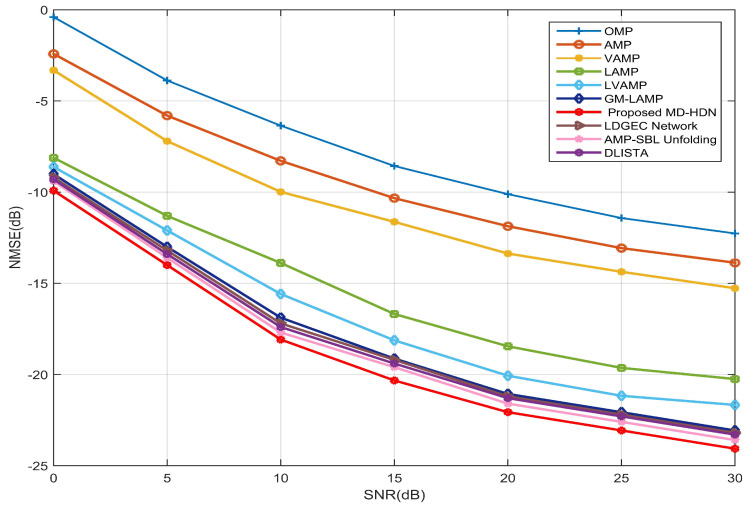
NMSE performance comparison between the proposed MD-HDN network and different algorithms with the UPA configuration under the Saleh–Valenzuela channel model.

Figure 7 further illustrates the NMSE performance of all considered channel estimation schemes as a function of SNR for the 16×16 UPA configuration. Conventional CS algorithms perform poorly, especially at low SNR, as their fixed priors cannot capture the two-dimensional angular clustering of beamspace channels. In contrast to the static priors employed in existing DL-based methods (e.g., AMP-SBL, GM-LAMP), the proposed MD-HDN dynamically refines the GMM parameters via the EM-GMM shrinkage function, thereby achieving superior estimation performance. At the SNR 30 dB, the MD-HDN outperforms the AMP-SBL by more than 1.5 dB, which confirms the effectiveness of the proposed scheme in complex planar array geometries.

### 4.3. Simulation Results on the DeepMIMO Dataset

In this subsection, we evaluate the wideband beamspace channel estimation performance of the proposed MD-HDN network against existing methods using the DeepMIMO dataset, which provides realistic channel realizations generated via ray tracing in a 3D environment.

**Figure 8:**
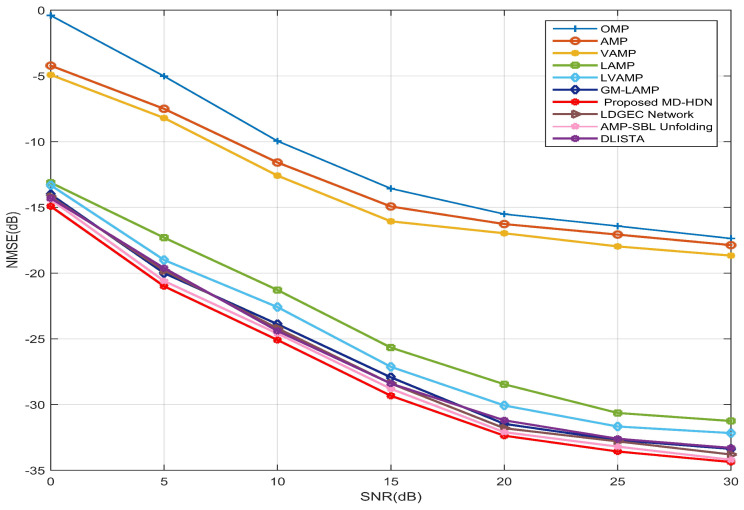
NMSE performance comparison between the proposed MD-HDN network and different algorithms with the ULA configuration based on the DeepMIMO dataset.

**Figure 9:**
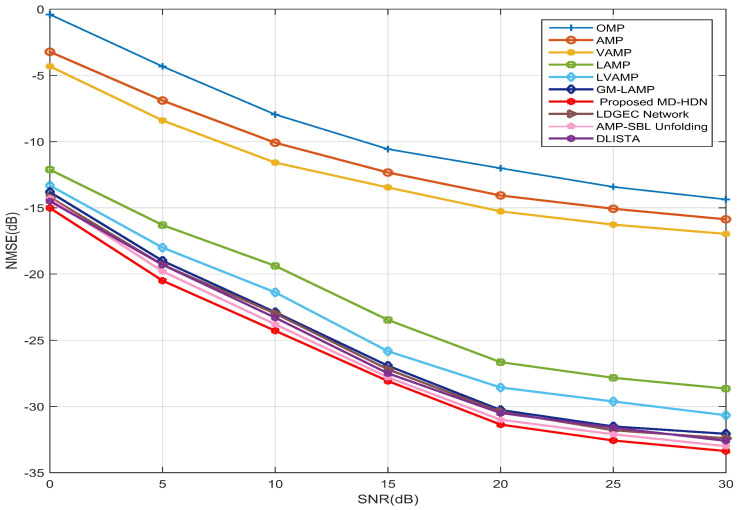
NMSE performance comparison between the proposed MD-HDN network and different algorithms with the UPA configuration based on the DeepMIMO dataset.

Figure 8 and Figure 9 illustrate the NMSE performance versus SNR for the 256×1 ULA and the 16×16 UPA, respectively. As shown in Figure 8, the proposed MD-HDN method demonstrates better estimation performance compared to both conventional CS algorithms (e.g., OMP, AMP, VAMP) and the DL-based network (e.g., LAMP, AMP-SBL, GM-LAMP) across all SNR levels in the ULA configuration. This advantage is attributable to capturing the sparse distribution of the wideband beamspace channel by means of the EM-GMM shrinkage function, thereby dynamically matching prior assumptions with the true channel statistics. Furthermore, Figure 9 shows that the proposed MD-HDN maintains its performance advantage under the more challenging UPA configuration. Compared to the higher-performing AMP-SBL scheme, the MD-HDN achieves a consistent performance gain of more than 1.6 dB at an SNR of 30 dB, highlighting the robustness of its iterative, prior-adaptive framework. These results confirm the generalization capability and robustness of the proposed scheme in practical mmWave massive MIMO systems.

**Figure 10:**
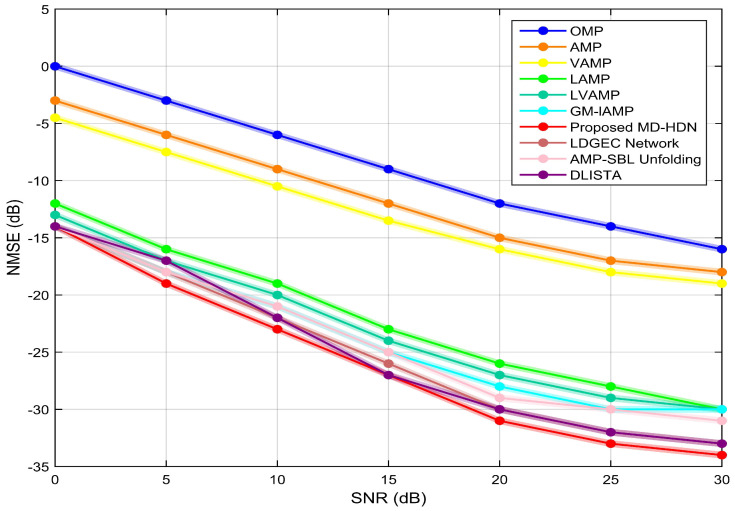
NMSE performance comparison of the proposed MD-HDN and baseline algorithms under the UPA configuration using the DeepMIMO dataset. Shaded regions denote ±1 sample standard deviation over 2000 channel realizations.

All the reported NMSE results are statistically averaged over 2000 independent channel realizations drawn from two complementary datasets, including the Saleh–Valenzuela model (capturing millimeter-wave channel characteristics) and the realistic DeepMIMO dataset (providing scenario-specific large-scale MIMO channel responses). To further quantify the statistical variability of the proposed data-driven method, the shaded regions in Figure 10 represent ±1 sample standard deviation across the 2000 trials. The standard deviation consistently remains at a low value (below 0.3 dB across all signal-to-noise ratio levels), thereby confirming that the proposed MD-HDN algorithms exhibit high reproducibility and are not attributable to random fluctuations.

The above experiments demonstrate that MD-HDN effectively mitigates the beam-squint effect in both ULA and UPA through the EM-GMM-based shrinkage function. This function adopts the GMM as the channel prior that inherently captures the clustered and sparse structure of the wideband beamspace channel, thereby yielding physically interpretable regularization. Notably, the GMM parameters are dynamically updated via the EM algorithm using the pilot observed on each subcarrier, which enables the estimator to track frequency-dependent shifts induced by beam-squint. In contrast, conventional deep unfolding methods, such as LAMP, LVAMP, and GM-LAMP, typically employ static or offline-trained shrinkage functions, which assume a common sparse structure across all subcarriers. These approaches fail to account for frequency variations, resulting in significant estimation errors and spectral energy leakage. Therefore, the proposed adaptive mechanism is pivotal to the MD-HDN, empowering it to achieve robust wideband beamspace channel estimation.

### 4.4. Other Simulation Results

**Figure 11:**
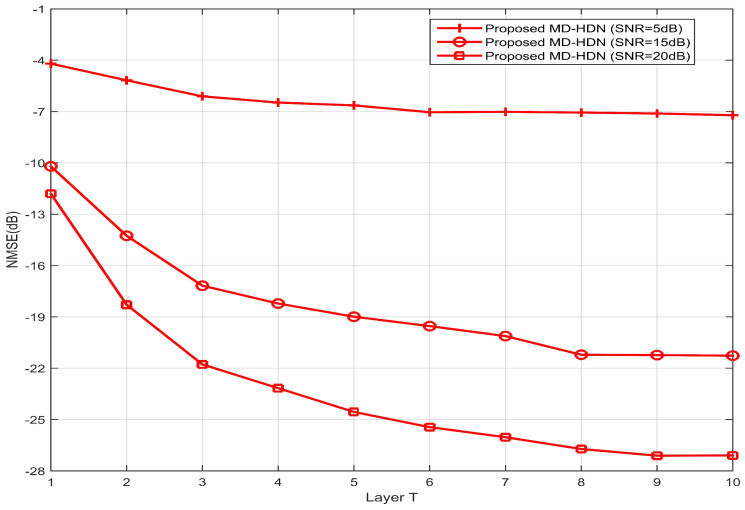
NMSE performance against the number of layers for the MD-HDN network with the ULA configuration based on the Saleh–Valenzuela channel model.

To investigate the convergence of the proposed MD-HDN scheme, we evaluate the NMSE performance as a function of the number of layers. A ULA configuration is considered and the Saleh–Valenzuela channel model is employed. As illustrated in Figure 11, the NMSE consistently improves with increasing layer depth under all SNR levels, which illustrates that the iterative unfolding structure of MD-HDN effectively enhances estimation accuracy. Specifically, convergence is achieved at approximately T=6 layers for the SNR =5 dB, whereas higher SNRs (15 dB and 20 dB) require about T=8 and T=9 layers, respectively. This SNR-dependent convergence behavior stems from the joint denoising and sparse support refinement performed across layers. Under low-SNR conditions (e.g., 5 dB), the estimation process is noise-dominated, and early saturation helps prevent overfitting to noisy measurements. In contrast, at high SNR, the stronger signal components allow subsequent layers to reliably resolve weaker multipath clusters, including their path gains, AoAs, and relative delays. Consequently, the network exploits additional layers to progressively refine the estimate, achieving higher reconstruction accuracy before converging at T=8 or 9. These results confirm that the proposed MD-HDN scheme can achieve an effective balance between convergence speed, estimation accuracy, and SNR adaptability, which makes it well suited for the sparse channel estimation in wideband beamspace systems. Following the convention for unfolded networks [23,27], the number of layers *T* serves as the primary trade-off between estimation accuracy and computational complexity. Other hyperparameters (e.g., the number of Gaussian components $N_{c}=4$) are fixed based on prior knowledge of mmWave channel sparsity.

**Figure 12:**
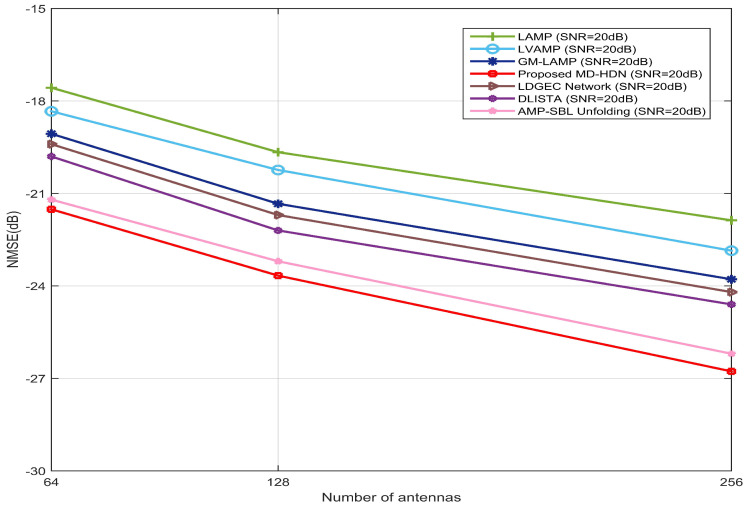
NMSE performance against the number of antennas between the proposed MD-HDN network and different algorithms with the ULA configuration based on the Saleh–Valenzuela channel model.

In Figure 12, the NMSE of several DL-based estimators is plotted against the number of antennas at SNR = 20 dB. As the array size increases from 64 to 256, all methods exhibit improved NMSE performance owing to the enhanced spatial resolution provided by larger antenna arrays. This improvement stems from the fact that a larger number of antennas enables finer separation of multipath components in the angular domain, thereby reducing estimation uncertainty.

Notably, by effectively exploiting prior distributions, the GM-LAMP and the proposed MD-HDN consistently outperform LAMP and LVAMP, respectively. In contrast, LAMP and LVAMP rely primarily on data-driven training without explicitly modeling the underlying sparsity or angular clustering characteristics. By incorporating learned priors, GM-LAMP and MD-HDN better capture the true channel distribution to perform support identification and amplitude estimation.

Among all methods, the proposed MD-HDN achieves the best performance across all antenna configurations, exhibiting a particularly pronounced gain at 256 antennas. These scalability advantages stem from its iterative architecture, which jointly refines channel support identification and noise suppression at each layer. As the array aperture increases, angular resolution improves, rendering previously inseparable weak multipath components resolvable. MD-HDN leverages this effect by progressively enhancing estimation fidelity through deeper unfolding, whereas other methods fail to fully exploit the additional spatial degrees of freedom. The sustained superiority of MD-HDN over unfolding-based baselines such as DLISTA and AMP-SBL further confirms its robustness to model mismatch and suitability for practical beamspace MIMO systems with varying array sizes.

**Figure 13:**
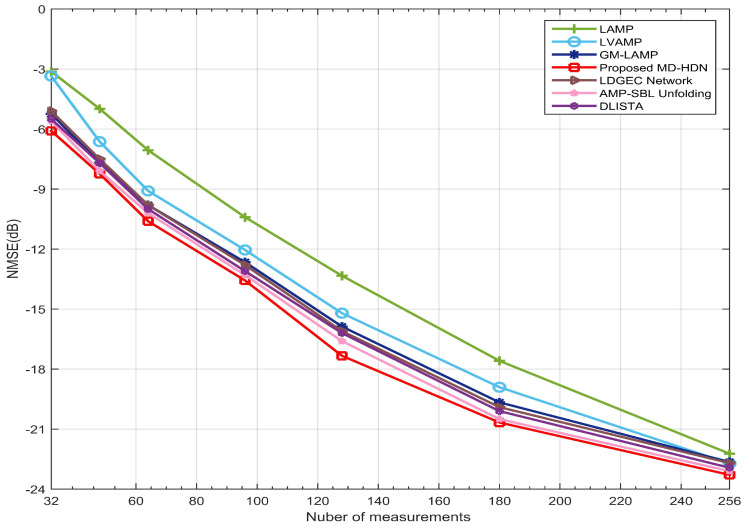
NMSE performance against the number of measurements for the proposed beamspace channel with the orthogonal pilot.

Figure 13 illustrates the NMSE performance as a function of the number of measurements. All methods achieve improved estimation accuracy as the number of measurements increases owing to enhanced signal recovery and reduced uncertainty. However, there are significant differences in their scalability, where LAMP, LVAMP, GM-LAMP, and LDGEC exhibit only gradual improvements. In contrast, the proposed MD-HDN method consistently maintains lower NMSE values across the entire measurement range and exhibits a steeper decline at higher measurement counts. This enhanced performance stems from the iterative unfolding architecture of MD-HDN, which adaptively optimizes channel support detection and magnitude estimation by leveraging newly acquired measurements. When measurements are limited, the network focuses on capturing dominant paths, while it resolves weaker components and refines phase and gain estimates. In contrast, unfolding-based methods such as DLISTA and AMP-SBL employ fixed optimization steps that cannot fully exploit the incremental information offered by additional measurements. Moreover, as the number of measurements increases, the NMSE curve of the proposed scheme declines more steeply, indicating that its adaptive learning mechanism exploits the acquired measurements more effectively.

**Figure 14:**
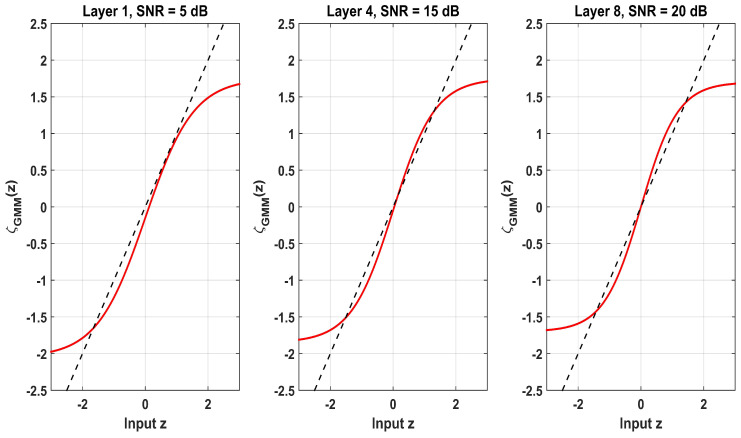
Learned shrinkage functions $\zeta_{\mathrm{GMM}}\left(z\right)$ at different layers and SNR levels.

Figure 14 shows the EM-GMM shrinkage functions $\zeta_{\mathrm{EM}\text{-}\mathrm{GMM}}\left(z\right)$ across different network depths and SNR levels. At Layer 1 with an SNR of 5 dB, the function exhibits a pronounced zero-attracting region that aggressively suppresses small-magnitude inputs to mitigate noise dominance. This reflects a conservative denoising strategy under poor channel conditions. In contrast, at Layer 8 with an SNR of 20 dB, $\zeta_{\mathrm{EM}\;\mathrm{GMM}}\left(z\right)$ closely follows the identity mapping y=z, which indicates that weak but reliable multipath components are preserved. The intermediate case Layer 4 with signal at an SNR of 15 dB exhibits a smooth transition between these two extremes. This layer- and SNR-dependent adaptation confirms that MD-HDN dynamically refines its GMM prior through data-driven learning to achieve an optimal trade-off between sparsity promotion and signal fidelity.

To provide a comprehensive and fair comparison among the DL-based network, inspired by the latency-weighted decoding framework [32], we introduce a latency-weighted NMSE metric that jointly considers both estimation accuracy and computational efficiency. Specifically, the latency-weighted NMSE is defined as

(35) $$L_{m}^{\rho }={\mathrm{NMSE}}_{m}^{\rho }\cdot T_{m},$$

where ${\mathrm{NMSE}}_{m}^{\rho }$ denotes the linear-scale NMSE of method *m* at SNR ρ, and $T_{m}$ is its inference latency normalized by that of the OMP algorithm.

**Figure 15:**
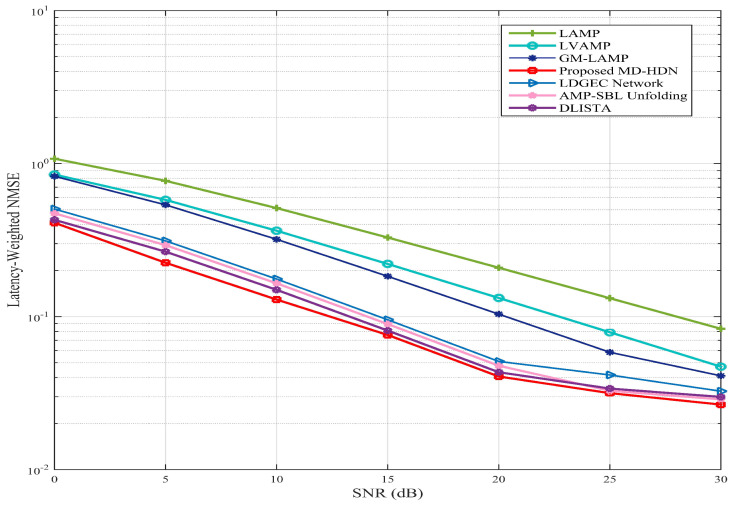
Latency-weighted NMSE versus SNR for various DL-based methods with the ULA configuration under the Saleh–Valenzuela channel model.

As shown in Figure 15, the proposed MD-HDN achieves the lowest latency-weighted NMSE across all SNR regimes, demonstrating its ability to strike an effective balance between estimation accuracy and computational complexity. At low SNR, the MD-HDN scheme outperforms GM-LAMP and LVAMP by approximately 15% in $L_{m}^{\rho }$, while maintaining a competitive advantage over LDGEC and AMP-SBL unfolding methods. As SNR increases, the performance gap widens owing to MD-HDN’s enhanced learning capability in exploiting sparse channel structures. While MD-HDN exhibits greater inference latency than lightweight models such as DLISTA, the corresponding reduction in NMSE yields a more favorable $L_{m}^{\rho }$, confirming that the accuracy-latency trade-off is well optimized for real-time massive MIMO systems.

## 5. Discussion

The model-data hybrid-driven scheme provides a robust and efficient solution to estimate the wideband beamspace channel estimation. It integrates domain knowledge such as structured sparsity and angular clustering into a learnable iterative framework, thereby combining the reliability of model-based methods with the flexibility of data-driven learning. By combining the reliability of physical channel models with the flexibility of data-driven learning, the hybrid approach delivers high estimation fidelity with limited training data and strong generalization capability, particularly well suited for 6G channel estimation, where dynamic propagation environments, ultra-large antenna arrays, and stringent spectral and energy efficiency requirements demand solutions.

## 6. Conclusions

In this paper, we propose a novel MD-HDN scheme for wideband beamspace channel estimation in mmWave Massive MIMO systems by integrating model-driven interpretability with data-driven adaptability. An EM-GMM shrinkage function is derived from the channel prior distribution and incorporated into an unfolded VAMP-based deep architecture to enhance estimation performance. Simulation results based on both the Saleh–Valenzuela model and the DeepMIMO dataset demonstrate that MD-HDN achieves superior estimation accuracy and robustness compared to existing methods across various SNR regimes and array configurations. Future work will extend the framework to multi-user and multi-cell scenarios, and explore the joint optimization of channel estimation and hybrid beamforming under highly dynamic or time-varying channel conditions envisioned in 6G systems.

## Figures and Tables

**Table 1:** Computational overhead comparison (M=256, N=64, T=10).

Method	Training Time (h)	Inference Time (ms)	Memory (GB)
OMP	–	0.9	0.3
AMP	–	1.4	0.5
VAMP	–	2.2	0.7
LAMP	3.2	7.0	0.9
LVAMP	5.1	8.3	1.1
GM-LAMP	6.0	9.7	1.3
MD-HDN (proposed)	**6.8**	**12.5**	**1.8**

**Table 2:** The channel parameters.

Channel Parameters	Value
Number of Paths (*L*)	3
Maximum Delay ($\tau_{max}$)	20 ns
Angle ($\theta_{l}$)	U(−π/2,π/2)
Complex gain ($\xi_{l}$)	CN(0,1)
Delay ($\tau_{l}$)	$U(0,\tau_{max})$

**Table 3:** The DeepMIMO dataset parameters.

Parameters	Value
Active BS	3
Number of Paths	3
Antenna spacing	0.5
Number of BS antenna	$\left(N_{x},N_{y},N_{z}\right)=\left(1,256,1\right);\left(1,16,16\right)$
Active user	From the row R1000 to R1300

## Data Availability

The original contributions presented in this study are included in the article. Further inquiries can be directed to the corresponding author.
